# Colorimetric Detection of Mercury Ions in Water with Capped Silver Nanoprisms

**DOI:** 10.3390/ma12091533

**Published:** 2019-05-10

**Authors:** Fouzia Tanvir, Atif Yaqub, Shazia Tanvir, Ran An, William A. Anderson

**Affiliations:** 1 Department of Zoology, Government College University, Lahore 54000, Pakistan; tanvir.fouzia@gmail.com (F.T.); atifravian@gmail.com (A.Y.); 2 Department of Chemical Engineering, University of Waterloo, Waterloo, ON N2L 3G1, Canada; shazia@genemis.ca (S.T.); r6an@uwaterloo.ca (R.A.)

**Keywords:** nanoparticles, nanoplates, spectral blue shift, amalgam, water quality

## Abstract

The emission of mercury (II) from coal combustion and other industrial processes may have impacts on water resources, and the detection with sensitive but rapid testing methods is desirable for environmental screening. Towards this end, silver nanoprisms were chemically synthesized resulting in a blue reagent solution that transitioned towards red and yellow solutions when exposed to Hg^2+^ ions at concentrations from 0.5 to 100 µM. A galvanic reduction of Hg^2+^ onto the surfaces is apparently responsible for a change in nanoprism shape towards spherical nanoparticles, leading to the change in solution color. There were no interferences by other tested mono- and divalent metal cations in solution and pH had minimal influence in the range of 6.5 to 9.8. The silver nanoprism reagent provided a detection limit of approximately 1.5 µM (300 µg/L) for mercury (II), which compared reasonably well with other reported nanoparticle-based techniques. Further optimization may reduce this detection limit, but matrix effects in realistic water samples require further investigation and amelioration.

## 1. Introduction

Mercury has been emitted by various industrial processes over the past century, with artisanal gold mining and coal combustion as the two largest sources currently, at 775 and 558 Mg per year respectively [1]. For coal combustion, just under 30% of these emissions are in the divalent form [1], Hg^2+^, which tends to have a high water solubility and shorter lifetime in the environment resulting in more local deposition [2]. As a monitoring tool, it is desirable to measure mercury content in aqueous samples to identify water sources that may be impacted by mercury emissions, since the US EPA limit for drinking water is 0.01 µM (2 ppb) [3]. The most common laboratory methods, using cold vapor atomic fluorescence spectrometry or atomic absorption spectrometry, are sensitive and accurate but require specialized facilities and equipment [4]. For screening purposes, it is desirable to have simple and rapid measurement techniques that can be used in the field, even if their sensitivity, accuracy and selectivity may not be as good as laboratory instrumentation. Therefore, a variety of other mercury detection methods have been developed over the years, ranging from colorimetric to electrochemical methods [5]. Molecular-based optical methods for heavy metal ion detection [6] have been developed and extended to mercury, including a wide range of chemical-binding and fluorescence detection schemes [7]. Other examples for the detection of aqueous phase mercury (II) include a nanoparticle-functionalized carbon paper electrode [8], a gold nanoparticle-aptamer colorimetric method [9], and a gold nanozyme paper chip [10].

Nanoparticle (NP)-based detection of aqueous phase cations has been targeted for a range of metal analytes, including nickel using glutathione-functionalized gold [11] or silver [12] NPs, lead using glutathione-functionalized gold nanostars [13], and cobalt using glutathione-modified silver NP spherical, plate and rod shapes [14]. The detection mechanism in these reports has been based on the induction of NP aggregation by the cations, resulting in a color or spectral change in the solution. Likewise, DNA-functionalized gold NPs have been shown to detect Hg^2+^ [15] through a similar aggregation mechanism.

Indeed, a variety of gold and silver nanoparticle applications for colorimetric Hg^2+^ detection has been a focus of research in recent years [16]. The aggregation or disaggregation of gold nanoparticles (AuNPs), functionalized or capped with various surface ligands, has been used to generate a spectral shift or color change in the presence of Hg^2+^ [16]. Silver nanoparticles (AgNPs) can likewise undergo a spectral shift due to aggregation, but the reduction of Hg^2+^ by Ag oxidation is also cited as a mechanism for color change [16]. A color change with silver nanoprisms, capped with 1-dodecanethiol, was induced by Hg^2+^ in the presence of added iodide anions and attributed to a morphological transformation in the nanoprisms [17]. Limits of detection for the nanoparticle-based methods vary widely depending on the specific formulation and measurement method (e.g. instrumental versus visual), but they tend to fall within the range of 10 nM to 55 µM mercury (II) ions for AgNPs [16].

A variety of different AgNP synthesis methods have been reported in the context of mercury (II) ion detection. Biological “green” synthesis has been used for AgNPs that shift in colour from yellow to colorless in the presence of mercury ions [18]. Some AgNP detection methods for mercury ion have employed various surface modifications including oligonucleotides [19], glutathione [20], and leaf extracts [21], for example. Chemically-synthesized AgNPs with a cytosine triphosphate cap demonstrated a similar decrease in yellow colour in the presence of mercury [22]. Another chemically-synthesized AgNP with a gelatin functionalization likewise showed a color change from yellow to colorless for Hg^2+^ concentrations as low as 25 nM [23]. A different color change, from orange to yellow, was created using AgNPs aggregated by the combination of mercury and lysine [24].

The majority of reports have been based on spherical AgNPs, and the blue-shift in absorbance generally results in the disappearance of the initial yellow color. A few researchers have used non-spherical nanoparticles such as one using triangular nanoplates assembled into thin films which resulted in a blue color [25]. A nanoprism formulation had a Hg^2+^ detection limit as low as 3.3 nM but required the presence of added iodide [17]. For visual detection, there are some advantages of having a more intense starting color such as the blue color attributed to silver nanoplates and nanoprisms, since the blue-shift caused by mercury may result in a more visible color change from blue to yellow or colorless.

Therefore, in this work, a simple chemical synthesis technique was adopted from earlier work with silver nanoprisms [26] to formulate non-spherical AgNPs with a visually-intense blue starting color. A straightforward capping method using polyvinylpyrrolidone was used to stabilize the AgNPs in solution in a form that may be more commercially suitable for shelf-life (versus DNA and similar functionalizations), and that avoided the need for other reagents such as iodide. The response of these AgNPs was tested to measure the spectral response to Hg^2+^ in water, and the sensitivity and response to other metal cations were determined. 

## 2. Materials and Methods 

Analytical grade (or of the highest purity available) chemicals were used. Solutions were prepared with ultra-pure water of typical resistivity 18.2 MΩ·cm. All chemicals were purchased from Sigma Aldrich (Canada) including sodium borohydride (NaBH_4_, 99.99%), hydrogen peroxide (H_2_O_2_ 30%), silver nitrate (AgNO_3_, 99.99%), trisodium citrate dihydrate (C_6_H_5_O_7_Na_3_ 2H_2_O, 99.99%), HNO_3_ and polyvinylpyrrolidone (PVP, Mw = 40,000), and the metal salts BaCl_2_, CdCl_2_, Cd(NO_3_)_2_, CoCl_2_·6H_2_O, CuSO_4_, FeCl_2_, HgCl_2_, KCl, K_2_CrO_4_, MgSO_4_, MnSO_4_, NaCl and Pb(NO_3_)_2_. The metal ion stock solutions were prepared by dissolving a measured amount of salts in 100 mL deionized water and diluting further as necessary. 

Silver nanoprisms were synthesized as follows. Sodium citrate (5.5 mM) was prepared in 100 mL deionized water, followed by adding 340 μL of AgNO_3_ (30 mM) and 560 μL H_2_O_2_ (30%). Then 2.3 mL NaBH_4_ (100 mM) was added and vigorously stirred. After 2 min, the colorless solution turned yellow and then rapidly darkened until a stable blue color was developed after 5 min. PVP was added at the final concentration of 0.03% as a capping agent to further stabilize the silver nanoprisms in solution. The synthesized AgNPs were stored in the dark and used as a stock solution [27]. The silver nanoprism solution absorbance was set by dilution to approximately 0.6 at 665 nm for use in mercury detection.

The synthesized silver nanoprisms were characterized using UV-visible spectrophotometry (HP 8542 Diode Array, Agilent Technologies, Santa Clara, CA, USA), energy-dispersive spectroscopy (EDX), transmission electron microscopy (TEM, Philips CM10, Amsterdam, The Netherlands), dynamic light scattering (DLS) and zeta potential. The colloidal solutions were centrifuged at 10,000 g and washed three times with deionized water. The washed samples were prepared by drying nanoparticles on a carbon tape. The elemental analysis of the nanoparticles was performed by energy dispersive X-ray (EDX) attached to the scanning electron microscope (model FEI/Philips XL30 FEG ESEM, Amsterdam, The Netherlands). The TEM samples were prepared by drop-coating the aqueous solution of nanoprisms onto a carbon coated copper grid (200 mesh), followed by air-drying for 2 h. TEM characterization was performed using a Philips CM10. 

A Zetasizer Nano ZS90 (Malvern Instruments Ltd, Malvern, UK) was used to measure particle size distribution by DLS, polydispersity index, and zeta potential, using triplicate runs with 10 measurements in each, and the Dispersion Technology software 5.1. For this analysis, a refractive index of 1.5 was used and the viscosity was assumed to be equal to that of the dispersant liquid.

The PVP capped nanoprisms were tested with various metal ions in aqueous solution with pH ranging from 5.8 to 9.7. The pH-dependent response was tested by dissolving the previously mentioned heavy metal salts in buffer (5 mM phosphate buffered saline). For the detection of Hg^2+^, solutions of different concentrations were mixed with the AgNP reagent in a 1:1 ratio in a buffer solution and left at room temperature for 30 min, after which the absorption spectra were measured. The comparison of the nanoparticle response to Hg^2+^ versus other metal ions (Ba^2+^, Cd^2+^, Co^2+^, Cu^2+^, Fe^2+^, K^+^, Mg^2+^, Mn^2+^, Na^+^, Pb^2+^, Zn^2+^) was investigated under the same conditions. EDTA (ethylenediaminetetraacetic acid) chelation was used as a negative control to confirm the reaction with mercury (II) by binding the metal ions in solution. The mercury ions were mixed with 10 mM of EDTA solution before the addition of the AgNPs.

## 3. Results and Discussion

### 3.1. Characterization of the Nanoparticles

Silver nanoprisms were prepared as described using the chemical reduction of silver nitrate to form nanospheres and a yellowish color in solution. This is followed by the transition of the solution to a blue color as the hydrogen peroxide induces anisotropic oxidation of portions of the nanoparticles into Ag^+^ ions, with a concurrent transformation of the spheres into nanoprisms [28]. 

**Figure 1:**
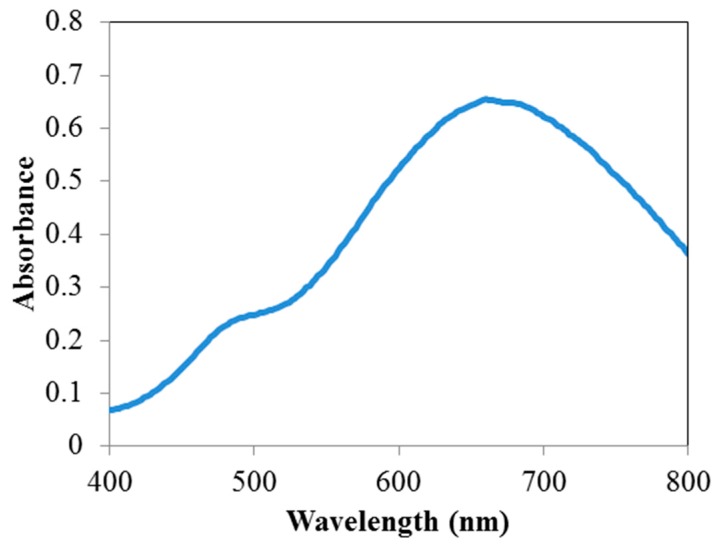
Absorbance spectrum of the as-prepared silver nanoparticle (AgNP) reagent solution before exposure to mercury (II) ion.

To verify the nature of the prepared colloidal suspension, the surface plasmon resonance (SPR) peak is seen in the absorbance spectrum (Figure 1). The spectral measurements of the colloidal solution clearly reflect a mixture of shapes since it features a small shoulder at around 450–475 nm (typical for nanospheres, with out-of-plane dipole resonance), and a more intense band at around 665 nm which has been reported previously for nanoprisms [26] with the in-plane dipole resonance of flatter shapes [29]. 

**Figure 2:**
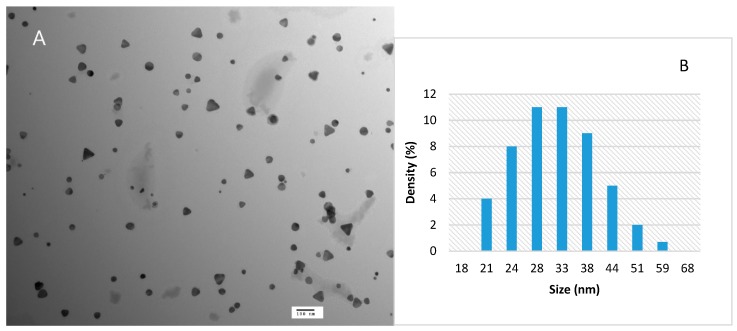
(**A**) TEM micrograph with 100 nm scale bar, and (**B**) particle size distribution profile of silver nanoparticles measured by dynamic light scattering (DLS).

**Figure 3:**
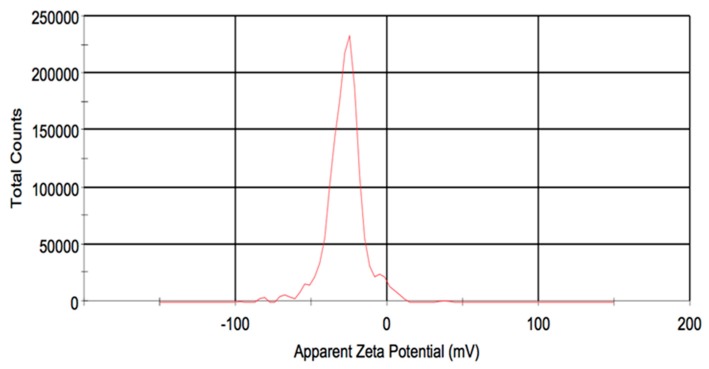
Surface charge distribution of Ag nanoprisms as measured by zeta potential.

The morphology suggested by the spectral measurement is verified by TEM imaging, where the AgNPs (Figure 2A) appear as a mixture of larger nanoprisms with a size of approximately 20 to 50 nm, and some smaller nanospheres, as was also shown in prior work using a similar synthesis method [26]. The particle size distribution profile was determined by DLS (Figure 2B), with an average size of 34.5 ± 6 nm, which was in reasonable agreement with the TEM results shown here and in other work. The polydispersity index reported by the instrument was lower than 0.3 in all cases, which is indicative of a relatively monodisperse system. The particles were determined to be negatively charged with a zeta potential of −27.6 ±2 mV (Figure 3).

Elemental analysis of the AgNPs was performed using EDX (energy-dispersive X-ray spectroscope). The EDX spectrum (not shown) clearly indicated that the prepared samples were pure silver with no contaminating substances other than a peak corresponding to carbon, which can be attributed to the carbon-coated tape sample substrate, and possibly the PVP capping agent.

### 3.2. Spectral Shifts in the Presence of Hg^+^

**Figure 4:**
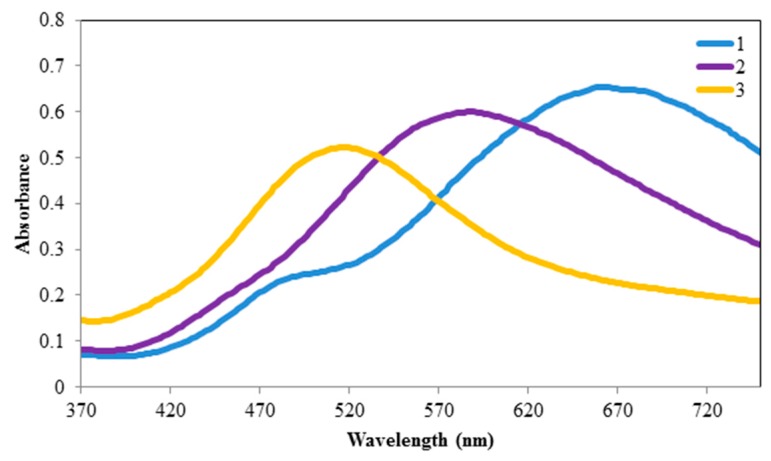
Absorbance spectra of the AgNPs in the absence (1, right) and presence of 2.5 µM (2, center) and 5 μM (3, left) HgCl_2_, after exposure for 30 min.

The AgNPs were used to detect Hg^2+^ using the shift of the maximum absorption wavelength after a 30 min incubation time. As shown in Figure 4, the AgNPs in the absence of mercury were characterized by a blue color and peak at 665 nm (curve 1). When Hg^2+^ was added (curves 2 and 3) the peak absorbance wavelength blue-shifted to shorter wavelengths, resulting in a more reddish visual color (see Figure 9 for color photograph). 

Thus, a blue-shift of the absorbance peak was observed in these experiments with a color change from blue (peak around 650 nm) to purple (peak around 530 nm) and eventually to yellow (peak around 450 nm) at high mercury concentrations. Some previous work has attributed the color shift to conformational changes in surface ligands, and/or aggregation of nanoparticles [15,19]. Other work has noted the reduction of Hg^2+^ onto the silver in the presence of H_2_O_2_ [20], and morphological changes to nanoprisms in the presence of iodide [17].

**Figure 5:**
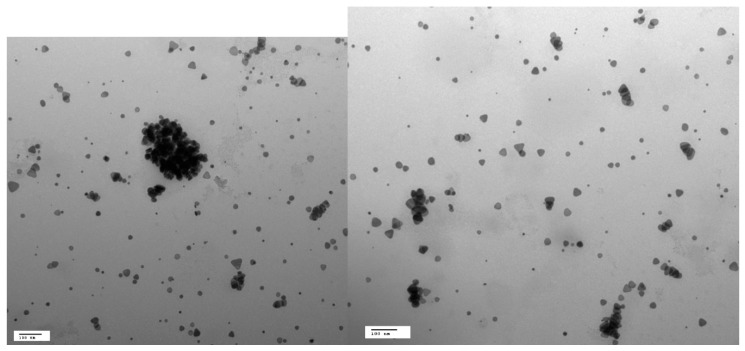
TEM images, with 100 nm scale bar, of AgNPs after reaction with 10 µM Hg^2+^ for 30 min.

In this work, there was some evidence for aggregation, as indicated in the TEM images in Figure 5. However, the induction of aggregation by the divalent mercury cation is difficult to reconcile with the lack of response to other divalent cations, as discussed below. However, as can also be seen in Figure 5, the corners of some of the prismatic AgNPs were rounded or possibly removed upon interaction with Hg^2+^, similar to morphological changes noted by others [17]. This resulted in a mixture of shapes, as illustrated in Figure 5, with relatively fewer and smaller prismatic shapes as compared to the image in Figure 2. These morphological changes and the lack of response to other cations (discussed below) suggest that aggregation did not play the only role in the spectral response and color change.

**Figure 6:**
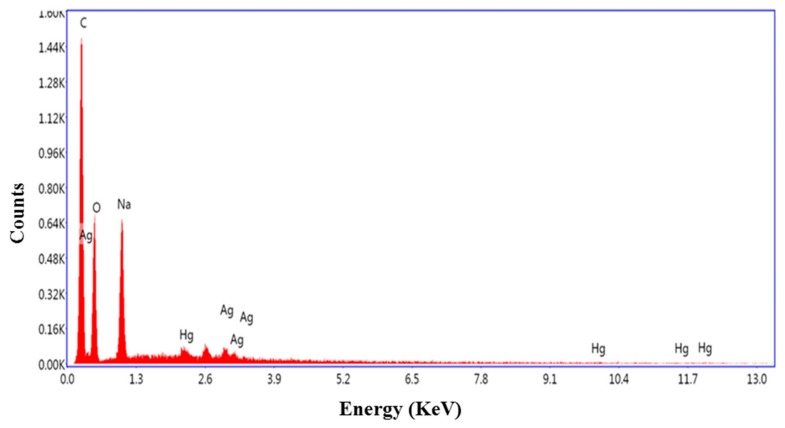
Elemental analysis, performed by energy dispersive X-ray spectroscopy (EDX), of Ag nanoparticles after exposure to mercury, indicating the formation of Ag-Hg amalgams.

The sharp edges of the nanoprisms are more likely prone to attack and in the presence of Hg^2+^ electrons could be initially extracted from the “corner” areas of the Ag nanoprisms resulting in the shape transformation. Unlike other reports of nanoprism morphology changes induced by Hg^2+^ [17], the response measured here did not require the addition of iodide and a thiol. The combination of silver or gold with mercury can lead to bimetallic colloids or amalgam formation, and this also leads to a blue shift of the absorption peak [30,31], although one report shows a slight red-shift in the presence of hydrogen peroxide [32]. An analysis of the AgNPs after exposure to mercury ion performed by EDX confirmed the presence of Hg in the colloids (Figure 6). Here, the Hg^2+^ detection appears to also be achieved by forming an Ag/Hg amalgam upon reduction of the mercury ion to elemental mercury by silver. The PVP AgNP capping agent may act as an electron donor, and the mercury reduction might also be supported by the PVP reduction abilities [33]. However, in the presence of the strong oxidizing agent (H_2_O_2_) used in the preparation of the nanoprisms, the continued presence of active reducing agents seems uncertain. Therefore, a galvanic replacement reaction, whereby a more reactive metal is dissolved and replaced by a less reactive one, seems more likely and this can occur rapidly at the nanoscale [34]. 

### 3.3. Sensitivity and Selectivity

**Figure 7:**
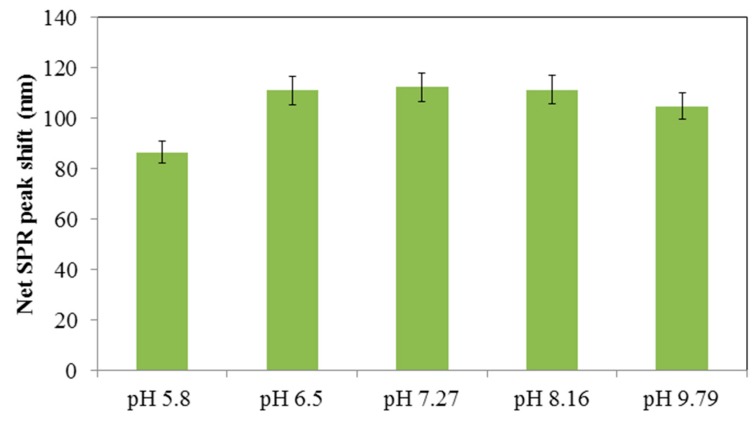
Influence of pH on the extent of the SPR peak shift at 5 µM of Hg^2+^ in 5 mM phosphate buffered saline.

The effect of pH on mercury detection was tested with 5 µM Hg^2+^ and the extent of the absorbance wavelength peak shift was quantified. As shown in Figure 7, the response of the AgNPs was relatively insensitive to pH ranging from 6.5 to 9.79, confirming that the PVP capped AgNPs exhibited excellent stability towards changes in pH in the neutral range [35]. A pH of 7.2 was selected to subsequently determine the sensitivity and selectivity since a commercialized test kit could readily incorporate a pH adjustment to neutral pH if necessary.

**Figure 8:**
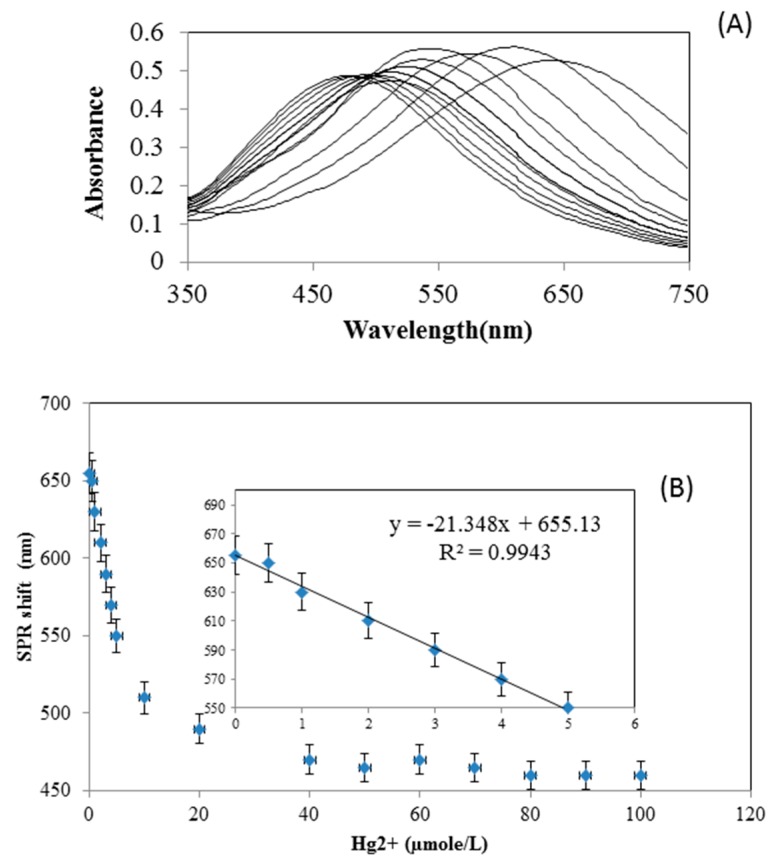
(**A**) UV−visible absorption spectra of the AgNPs after the addition of Hg^2+^ at various concentrations, with the peak shifting from the right (for 0 µM) to left as Hg^2+^ concentration increases up to 100 µM. (**B**) Peak wavelength of the AgNPs at varying concentrations of Hg^2+^ (0−100 µM), where the inset graph shows a linear regression for the range of 0 to 5 µM. The error bars show standard deviations (three measurements).

To evaluate the sensitivity of the nanoprisms to Hg^2+^ concentration, the absorption spectra of the AgNP solutions were measured under the optimized conditions, using different Hg^2+^ concentrations added to the solution, with incubation for 30 min. Figure 8A shows that increasing concentrations of Hg^2+^ resulted in a larger blue shift of the absorbance peak, as attributed to the morphology changes illustrated earlier. Therefore, the extent of the peak shift could potentially be employed as a quantitative analysis for Hg^2+^. This change in peak wavelength versus Hg^2+^ concentration (0.5–100 µM) relationship is shown in Figure 8B. A linear relationship was obtained over the range of 0 to 5 µM with a correlation coefficient of 0.993 (Figure 8B inset). The results indicate that this AgNP material could be used to detect Hg^2+^ at a detection limit of approximately 1.5 µM (approximately 300 µg/L), based on three standard deviations. Although not the most sensitive detection limit ever reported, it compares favorably with the range of 10 nM to 55 µM for other AgNPs reported in one review [16]. The standard deviations shown in Figure 8B suggest that the AgNPs may not be suitable for accurate quantification of Hg^2+^ at lower concentrations, but they may serve the purpose of rapid screening and “yes/no” detection of Hg^2+^ above some minimum threshold. Further improvements in sensitivity may be possible with optimization of the concentrations and ratios of AgNPs to sample solutions.

**Figure 9:**
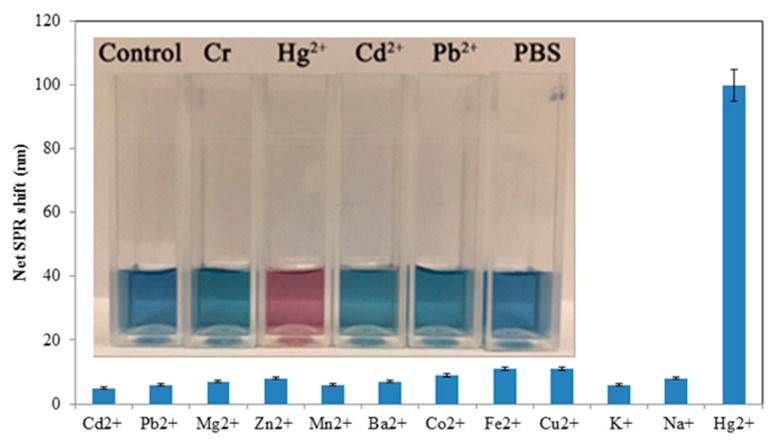
Effect of various metal ions (5 µM) dissolved in 5 mM phosphate buffered saline pH 7.2 on the net SPR shift, showing high reactivity with only Hg^2+^. Inset: photograph of AgNP solutions, showing color change with Hg^2+^ and lack of significant response to phosphate-buffered saline (PBS), deionized water (Control), and the other ions Cd^2+^, Pb^2+^ and CrO_4_^2-^ (labelled “Cr”) at 10 µM.

If a galvanic replacement reaction between Hg^2+^ and Ag^0^ is responsible for the detection mechanism and not just aggregation, as discussed earlier, then the AgNPs should not be reactive towards other cations in solution. To determine and verify the response of the nanoprisms towards Hg^2+^, the effects of Ba^2+^, Cd^2+^, Co^2+^, Cu^2+^, Fe^2+^, K^+^, Mg^2+^, Mn^2+^, Na^+^, Pb^2+^, and Zn^2+^ were determined individually under the selected test conditions. The results shown in Figure 9 indicated that Hg^2+^ alone caused a significant effect on the silver nanoparticles, as determined by spectroscopy (some examples of which color are shown in the Figure 9 inset). A 100-fold higher concentration of the other metal ions also had no significant effect on the SPR peak shift (not shown). These results indicate that the AgNPs exhibited a very high reactivity toward Hg^2+^ ions only, and provide further evidence that the interaction between the AgNPs and Hg^2+^ is primarily driven by a galvanic reaction. The standard reduction potentials for Hg^2+^ and Ag^+^ are 0.85 and 0.80 V, respectively, while those of the other species tested here are all <0.80 V [36]. Therefore, of all these metals only Hg^2+^ can potentially be reduced by silver, lending support to the hypothesis that a galvanic reaction is primarily responsible for the nanoprism morphology changes and SPR blue-shift, rather than aggregation effects alone. There are very few, if any, metals of environmental significance with a reduction potential >0.80 V, suggesting that the silver nanoprisms will always be selective towards mercury, in the absence of any other interfering effects that remain to be discovered. Mixtures of the other metal cations and Hg^2+^ were not tested but should be in future work to ensure that surface complexation by other metals will not inhibit or affect the AgNP interactions. Likewise, since these experiments were carried out with HgCl_2_ only, the effects of other counter-ions such as nitrate would be of interest to explore.

To further explore the nature of the nanoprism/metal interactions, negative control experiments were performed using the metal ion chelator EDTA. The various metal ions and Hg (II) were mixed with 10 mM EDTA and then allowed to interact with the prismatic silver reagent for 30 min. No color change was noted under these conditions, confirming that the previously measured effects are due to free Hg^2+^ and that chelated ions may not be detectable via this method.

**Figure 10:**
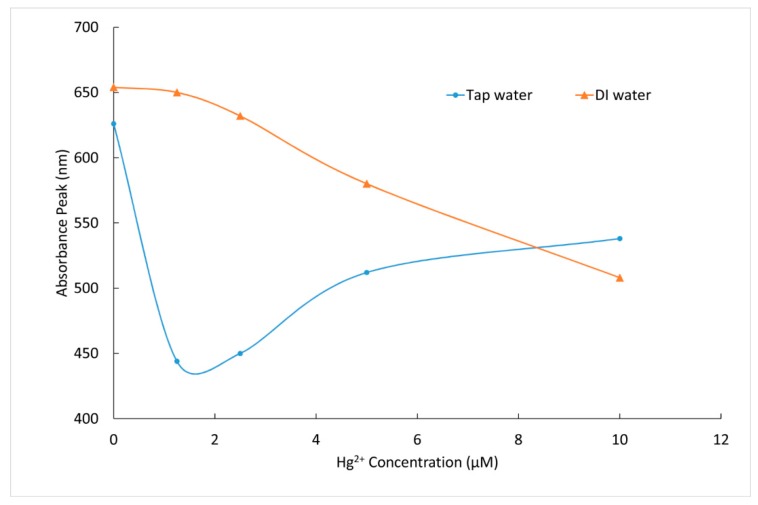
Silver nanoprism solution absorbance peak as a function of Hg^2+^ concentration in deionized (DI) water (upper curve) and municipal tap water (lower curve), showing non-linear blue-shift in the tap water matrix.

Furthermore, some tests were completed in 0.2 µm filtered municipal tap water to assess the potential interference from other typical water species, with the results shown in Figure 10. Unlike the linear response in pure water and buffer solutions, AgNP responses in the tap water matrix showed a complex non-linear response to increasing Hg^2+^ concentrations. Further work remains to be pursued to determine the reasons behind this difference, but the presence of the oxidant hypochlorite (approximately 0.5 mg/L), alkalinity, or organic carbon such as humic substances may play a role. Matrix effects continue to be a challenge for many nanoparticle-based detection methods [37].

## 4. Conclusions

The technique explored in this study, using PVP-capped prismatic silver nanoparticles, provides a rapid, reasonably sensitive and very specific detection method for aqueous Hg^2+^ samples and may be potentially suitable for remote field and environmental analysis where more advanced instrumentation is not readily available. A galvanic reduction of Hg onto the silver leads to a loss of prismatic size and shape and a blue-shift in the absorbance spectrum, as suggested by TEM, the EDX spectrum, and the lack of sensitivity to other metal ions. The minimum detection limit for this reagent was found to be approximately 1.5 µM under controlled conditions, which may be suitable for environmental screening purposes but not for drinking water testing which requires a 0.01 µM or lower detection limit. The response of the AgNPs to Hg^2+^ alone was confirmed through experiments with a variety of other common metals ions in solution. Interactions with other cations and anions may alter the response to Hg^2+^, as shown in testing with a municipal water sample and this requires further study to identify the interference mechanisms. Using these prismatic silver nanoparticles may offer a useful approach for the detection of Hg^2+^ in aqueous environmental samples, but additional optimization work is required to lower the detection limit further and to ascertain how to minimize water matrix effects.
